# Transpapillary Drug Delivery to the Breast

**DOI:** 10.1371/journal.pone.0115712

**Published:** 2014-12-29

**Authors:** Kaushalkumar Dave, Ranjith Averineni, Preety Sahdev, Omathanu Perumal

**Affiliations:** Department of Pharmaceutical Sciences, South Dakota State University, Brookings, South Dakota, 57007, United States of America; University of Quebec at Trois-Rivieres, Canada

## Abstract

The study was aimed at investigating localized topical drug delivery to the breast via mammary papilla (nipple). 5-fluorouracil (5-FU) and estradiol (EST) were used as model hydrophilic and hydrophobic compounds respectively. Porcine and human nipple were used for in-vitro penetration studies. The removal of keratin plug enhanced the drug transport through the nipple. The drug penetration was significantly higher through the nipple compared to breast skin. The drug’s lipophilicity had a significant influence on drug penetration through nipple. The ducts in the nipple served as a major transport pathway to the underlying breast tissue. Results showed that porcine nipple could be a potential model for human nipple. The topical application of 5-FU on the rat nipple resulted in high drug concentration in the breast and minimal drug levels in plasma and other organs. Overall, the findings from this study demonstrate the feasibility of localized drug delivery to the breast through nipple.

## Introduction

Breast cancer is the second leading cause of cancer related deaths in women [1]. Majority of breast cancers originate from the epithelial cells lining the milk ducts [2]. Although, most breast cancers are localized to the breast, some can progress to invasive, metastatic breast cancer [2]. Ductal carcinoma in-situ (DCIS) is a common breast cancer in women, where localized therapy can be most beneficial. Further, localized therapy can be an adjuvant to other systemic therapies in breast cancer. The current treatment options for breast cancer include mastectomy, breast conserving surgery (lumpectomy), radiation therapy, hormone therapy, chemotherapy and biological therapy [2], [3]. However, these treatments are associated with short-term and long-term side effects including cardiac complications, risk of second cancers, anemia, peripheral neuropathy, myelosuppression, thromboembolism and psychological distress [4]–[6]. Hence, there is a strong need to develop safe therapeutic approaches for breast cancer.

With the recent developments in ductoscopy, there has been interest in developing localized therapeutic approaches for breast cancer [7]. The intraductal injection of anti-cancer drugs has been reported to show better efficacy than intravenous (i.v.) injection in animals [7]–[9]. Further, in breast cancer patients, the intraductal injection showed higher drug levels in the breast and lower drug levels in the blood [8]. However, the intraductal injection is an invasive procedure that requires significant expertise and skill to identify a single duct for injection of anti-cancer agents. On the other hand, the topical application of anti-cancer agents on the breast skin has also been explored as a localized therapeutic approach [10]–[13]. However, the poor skin permeability results in limited drug concentration in deeper breast tissue [10]–[13]. To this end, the unique anatomy of the mammary papilla (nipple) provides a potential route for direct drug delivery to the underlying breast tissue. The multiple duct openings on the surface of the nipple directly extend into the terminal duct lobular units of the breast [14]. Furthermore the nipple-areola complex has a thinner epidermis compared to the skin [15]. The nipple-areola complex also has abundant appendages including apocrine, sebaceous and eccrine sweat glands, all of which can serve as potential transport pathways to the underlying breast tissue [16], [17]. Recently, Lee et al demonstrated the in-vitro delivery of delivery of anti-cancer compounds through porcine nipple [18]. However, the factors that influence drug transport through the nipple is yet to be established. To this end, the objective of this study was to investigate the influence of lipophilicity on molecular transport through the nipple. The second goal was to compare the in-vitro drug permeability between skin and nipple in human and porcine breast tissue. Finally, the goal was to test if localized topical drug delivery can be achieved in-vivo in rats.

## Materials and Methods

### Ethics Statement

The human breast tissue used in the study was collected from human cadavers and is an exempt protocol as per the guidelines of Institutional Review Board at South Dakota State University. The animal studies were approved by the Institutional Animal Care and Use Committee at South Dakota State University (Approval Number: 12-029A).

### Materials

5-Fluorouracil (5-FU), estradiol (EST) and sulforhodamine B (SRB) were purchased from Sigma-Aldrich, St. Louis, MO. Nile Red (NR) was purchased from MP Biomedicals, Solon, OH. ^14^C-5-FU and ^3^H-EST were purchased from Moravek Biochemicals and Radiochemicals, Brea, CA. Biosol (tissue solubilizer), Bioscint and Ecoscint (scintillation cocktails) were purchased from National Diagnostics, Atlanta, Georgia. Isoflurane was purchased from VetOne, Boise, ID. All other reagents were purchased from Sigma-Aldrich, St. Louis, MO, USA.

### Tissue preparation

Porcine tissue was procured from the slaughterhouse in the Department of Animal Science at South Dakota State University (Brookings, SD, USA). Nipple and breast skin were collected from 6–8 months old female pigs and was washed under tap water. Breast tissue from human cadavers was procured from the National Disease Research Interchange (Philadelphia, PA, USA) and South Dakota Lions Eye and Tissue Bank (Sioux Falls, SD, USA). The abdominal fat underneath the breast tissue was removed using a scalpel. For skin penetration studies, the breast skin surrounding the nipple was collected and dermatomed to 700 µm thickness (Padgett Instruments, St. Louis, MO, USA).

### *In-vitro* penetration studies

**Figure 1:**
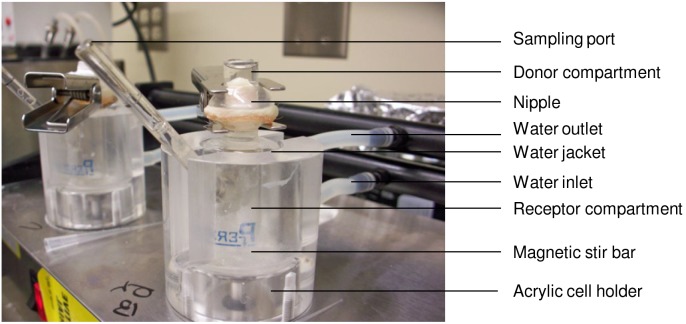
Experimental set-up for in-vitro drug penetration study.

The nipple (Fig. 1) or the breast skin was sandwiched between the two compartments of a vertical Franz diffusion cell (0.63 cm^2^; PermeGear, Hellertown, PA, USA). The nipple was used as such or after removing the keratin plug by wiping the surface of the nipple using 70% alcohol. The removal of the keratin plug was confirmed using a stereo-microscope (Figure S1 in S1 File). In this study, 5-fluorouracil (5-FU; MW = 130 Da; Log P = −0.89) and estradiol (EST; MW = 273 Da; Log P = 3.6) were used as model hydrophilic and hydrophobic drugs respectively. The receptor medium was composed of phosphate buffer containing 0.05% w/v of sodium azide (pH 7.4) for 5-FU and ethanol: phosphate buffer (20∶80) for EST. The receptor compartment was maintained at 37°C and stirred using a magnetic bar. Saturated solution (500 µl) of 5-FU (spiked with 0.25 µCi ^14^C-5-FU) and EST (spiked with 0.5 µCi ^3^H-EST), prepared in ethanol: water (50∶50), was applied in the donor compartment. The study was performed for 6–48 hrs and the samples (200 µl) were collected from the receptor compartment at different time points. At the end of the study, the tissue was removed and digested using tissue solubilizer (Biosol). The samples were mixed with 2 ml of scintillation cocktail (Ecoscint was used for receptor compartment samples and Bioscint was used for tissue homogenate samples) and the radioactive counts were measured using a liquid scintillation counter (Beckman Coulter LS6500).

### Tissue histology

For studying the histology of porcine nipple, the tissue was fixed in 10% buffered neutral formalin for 72 hrs at room temperature. Then, the tissue was embedded in paraffin and 5 µm thick cross sections were prepared using a rotary microtome (Olympus CUT 4060E, Center Valley, PA, USA). The sections were stained with hematoxylin-eosin and examined under a light microscope (Olympus AX70, Center Valley, PA, USA).

### *In-vitro* penetration study using fluorescent dyes

To determine drug transport pathways in human/porcine nipple, SRB and NR were used as model hydrophilic and model hydrophobic fluorescent dyes respectively. SRB (10 mM) in 1∶1 of ethanol: phosphate buffer and NR (0.32 mM) in propylene glycol was applied in the donor compartment for 12 hrs. At the end of the study, the tissue was removed, washed and analyzed by microscopy.

### Confocal laser scanning microscopy (CLSM)

At the end of the dye penetration studies, 1 mm thick cylindrical section was cut from the middle of the porcine nipple (∼5–6 mm from the top of the nipple) and placed on a glass slide. The tissue sections were observed in a confocal microscope (Fluoview FV300, Olympus ix70, Olympus, Center Valley, PA, USA). The fluorescent dyes were excited using a green helium-neon laser at an excitation wavelength of 543 nm. Confocal images were obtained in the xy plane and xz plane. For the xz sections, a horizontal line was defined across the region of interest in the z = 0 µm in xy plane and then optically sectioned from the surface of the tissue (z = 0 µm) to 500 µm depth at a step size of 5 µm/scan. For xyz images, the tissue was scanned from the surface (z = 0 µm) to 500 µm depth at a step size of 25 µm/scan.

### Fluorescence microscopy

To understand the depth of penetration, dye distribution in different sections from the tip to the base of the nipple was studied. The tissue was snap frozen in optimum cutting temperature (OCT, Tissue Tek, Torrance, CA, USA) medium placed in hexane under liquid nitrogen. The OCT block was used to prepare 7–8 µm thick sections in a cryostat (UV800, Leica Microsystems, Bannockburn, IL, USA) at −25°C. The section was dried for 6 hrs at 37°C and the cover slip was placed over the section and sealed using CytoSeal (Vector laboratories, Burlingame, CA, USA). The tissue sections were observed under a fluorescence microscope (Olympus AX70, Center Valley, PA, USA).

### *In-vivo* studies

**Figure 2:**
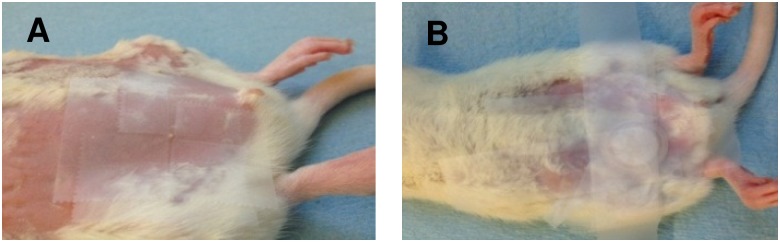
Experimental set-up for topical application of 5-FU on nipple in-vivo. (A) Adhesive tape was applied around nipple to limit the topical drug delivery through the nipple; (B) Hilltop chamber was used for topical drug application.

For in-vivo studies, 7–10 week old female Sprague-Dawley rats (Charles River Laboratories, Wilmington, MA, USA), were used. The experimental protocol was approved by the Institutional Animal Care and Use Committee. For topical/transdermal drug application, the rat abdominal hair was removed two days before the study using a hair clipper and depilatory cream (Nair Hair Remover Lotion, Princeton, NJ, USA). For topical delivery through the nipple, the surrounding breast skin was covered with scotch tape (3M, St. Paul, MN, USA) to limit drug exposure to the nipple (Fig. 2A). Keratin plug was removed from the surface of the nipple using 70% alcohol. Around 250 µl of 5-FU solution (10 mg/ml of 5-FU in 50% ethanol spiked with 0.25 µCi of ^14^C-5-FU) was loaded in a Hill Top chamber (3.8 cm^2^; Hill Top Research, St. Petersburg, FL, USA) and applied on the nipple or on the breast skin surrounding the nipple (Fig. 2B). The drug was applied for 2–6 hrs under Isoflurane anesthesia (0.75% isoflurane and oxygen flow rate of 0.8 L/min; VetEquip-VE 2848, Pleasanton, CA, USA). In a separate study, to understand the drug disposition from the breast tissue, the treatment was removed from the breast after 6 hrs and the study was continued for another 6 hrs. For intravenous drug administration, 5-FU solution (10 mg/ml of 5-FU spiked with ^14^C-5-FU) was prepared in 0.9% NaCl and 50 µl was injected into the tail vein using 27-gauge, ½, needle (BD, Franklin Lakes, NJ, USA).

Blood samples were collected at the end of the study from the retro-orbital plexus under mild isoflurane anesthesia. The blood samples were collected into heparinized Eppendorf tubes and then centrifuged at 10,000 rpm for 15 minutes. After centrifugation, plasma was collected and mixed with scintillation cocktail (Bioscint). At the end of the study, the animals were euthanized by CO_2_ asphyxiation. Before collecting the organs, the tissue was perfused with saline to remove blood from the organs. The nipple, mammary gland, kidneys, liver, spleen, lungs, brain and heart were collected. The tissue was weighed and homogenized (Omni General Laboratory Homogenizer, Omni International, Kennesaw, GA, USA). The homogenate was then mixed with tissue solubilizer (Biosol), and incubated at 50°C for 24 hrs. The sample was quenched using 30% v/v hydrogen peroxide (H_2_O_2_) solution and incubated at 50°C for 4 hrs. The sample was then mixed with scintillation cocktail (Bioscint) and the radioactive counts were measured using a liquid scintillation counter.

### Data analysis

The cumulative amount of drug permeated per unit area of the tissue was plotted against time. Flux was obtained from the slope of linear portion of the curve and lag time was calculated by extrapolating the linear portion of the curve to the x-axis (time). Cumulative amount of drug permeation through the tissue and drug retention in the tissue were also calculated. All the experiments were performed in triplicate or quadruplicate and the results were expressed as mean ± SD. Two-tail unpaired *t*-test and one-way ANOVA test (Instat 3, GraphPad software, CA, USA) were used to compare the treatment groups and the results were considered to be significant at p<0.05.

## Results

### *In-vitro* penetration of hydrophilic model drug

**Figure 3:**
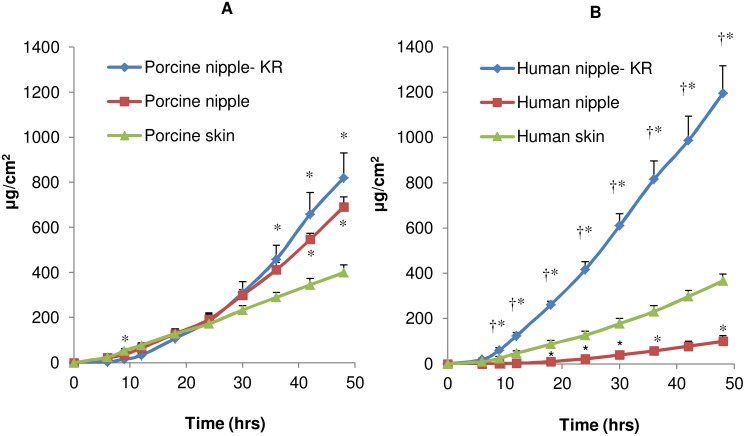
Permeation of 5-FU through excised breast tissue. In-vitro permeation of 5-FU through porcine (A) and human (B) nipple after removing the keratin plug (KR), nipple with keratin plug, and breast skin. Each data point is represented as mean ± SEM (n = 3–4). † is significant in comparison to the results from nipple with keratin plug; *is significant in comparison to skin. The values are significant at p<0.05, by one-way ANOVA.

For this study, 5-FU, a drug used in breast cancer was chosen as a model hydrophilic molecule [19]. The drug transport across the nipple into the receptor medium in-vitro is representative of the drug penetration into the underlying breast tissue in-vivo. The in-vitro penetration studies were conducted on the nipple without any treatment or after removal of the keratin plug using 70% alcohol. As shown in Fig. 3, the 5-FU penetration increased with time and there was no significant effect of keratin plug on 5-FU penetration through the porcine nipple (Fig. 3A). However, 5-FU penetration through the porcine nipple was higher compared to the drug penetration through the breast skin. The steady state flux and cumulative amount of 5-FU penetration through the nipple was 2–3 fold higher compared to breast skin. As seen from the lag time values (Table 1), the drug transport through the nipple was slower than through the breast skin. On the other hand, drug retention in the nipple was comparable to drug retention in the breast skin.

**Table 1:** In-vitro penetration parameters of 5-FU in porcine and human tissue.

Treatment Group	L (hrs)	J_ss_ (µg/cm^2^/hrs)	Q_48_ (µg/cm^2^)	C_T_ (µg/mg)
Porcine nipple-keratin plug removed	13.02±0.83^a^	24.87±4.39^a^	1041.36±184.01^a^	1.24±0.05
Porcine nipple	14.27±1.61^a^	19.25±0.80^a^	691.90±43.09	1.44±0.17
Porcine breast skin	4.01±0.51	8.80±0.58	399.82±33.71	0.90±0.18
Human nipple-keratinplug removed	7.91±0.91^b^	27.05±1.03^bc^	1195.95±121.47^bc^	1.41±0.36^b^
Human nipple	19.17±2.34^c^	3.38±0.66	100.07±23.76	0.41±0.11
Human breast skin	5.69±0.92	6.47±0.78	367.20±29.73	1.11±0.03

L is lag time; J_ss_ is flux at steady state; Q_48_ is cumulative amount of drug permeated per cm^2^ of the tissue at 48 hrs; C_T_ is tissue drug amount at 48 hrs. Results are presented as mean ± SEM (n = 3–4); ‘a’ is significant in comparison to porcine skin; ‘b’ is significant in comparison to human nipple with keratin plug; ‘c’ is significant in comparison to human skin. The values are significant at p<0.05 by one-way ANOVA.

In contrast to porcine nipple, keratin plug significantly reduced 5-FU penetration through human nipple (Fig. 3B; Table 1). In presence of keratin plug, the 5-FU penetration was lower than the breast skin. The lag-time was significantly shorter after removing keratin plug from human nipple (Table 1). Similar to porcine tissue, the flux and cumulative amount of 5-FU penetration was higher compared to human breast skin (Table 1). After removing the keratin plug, the permeation of 5-FU through the porcine nipple was relatively closer to the permeation of 5-FU through human nipple.

### *In-vitro* penetration of hydrophobic model drug

**Figure 4:**
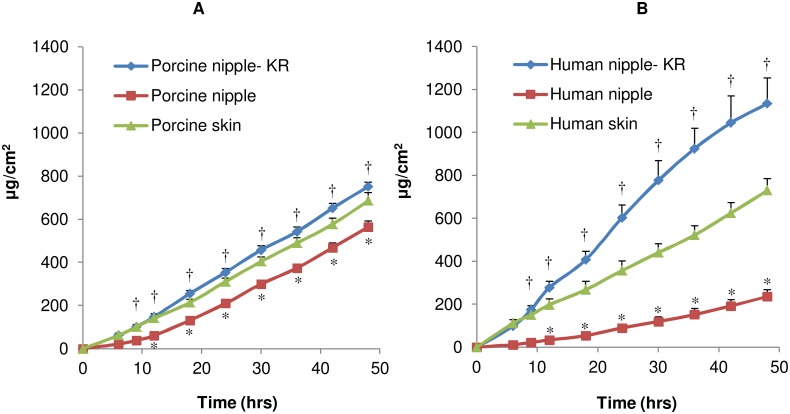
Permeation of EST through excised breast tissue. In-vitro permeation of EST through porcine (A) and human (B) nipple after removing keratin plug (KR), nipple with keratin plug, and breast skin. Each data point is represented as mean ± SEM (n = 3–4). † is significant in comparison to the results from nipple with keratin plug; *is significant in comparison to corresponding skin group. The values are significant at p<0.05, by one-way ANOVA.

Estradiol (EST) was used as a model hydrophobic molecule and this drug was chosen due to its similar physicochemical properties to anti-estrogen compounds used in breast cancer [20]. In general, the lag-time was shorter for EST compared to 5-FU (Tables 1 and 2). In porcine tissue, the EST penetration was similar between nipple and breast skin (Fig. 4A). The keratin plug had no significant effect on the EST flux in porcine nipple (Table 2). However, after removing the keratin plug, the lag-time reduced significantly (Table 2). The cumulative amount of EST penetrated through the porcine nipple was higher after removing the keratin plug.

**Table 2:** In-vitro penetration parameters of EST in porcine and human tissue.

Treatment Group	L (hrs)	J_ss_ (µg/cm^2^/hrs)	Q_48_ (µg/cm^2^)	C_T_ (µg/mg)
Porcine nipple-keratinplug removed	2.71±0.52^ab^	16.69±0.62	752.76±19.58^a^	1.48±0.16
Porcine nipple	9.73±0.81^b^	14.49±0.55	564.55±36.16^b^	0.84±0.06^b^
Porcine breast skin	4.99±0.15	15.90±0.54	686.40±27.27	5.69±0.28
Human nipple-keratinplug removed	2.15±0.09^c^	27.25±2.63^c^	1135.30±118.93^cd^	2.21±0.18^cd^
Human nipple	9.13±1.82^d^	5.62±0.76^d^	236.73±29.85^d^	0.37±0.09^d^
Human breast skin	0.63±0.24	18.38±2.91	730.81±54.31	0.81±0.06

L is lag time; J_ss_ is flux at steady state; Q_48_ is cumulative amount of drug permeated per cm^2^ of the tissue at 48 hrs; C_T_ is tissue drug amount at 48 hrs. Results are presented as mean ± SEM (n = 3–4); ‘a’ is significant in comparison to porcine nipple with keratin plug; ‘b’ is significant in comparison to porcine skin; ‘c’ is significant in comparison to human nipple with keratin plug; ‘d’ is significant in comparison to human skin. The values are significant at p<0.05 by one-way ANOVA.

Similar to 5-FU, the keratin plug had a significant influence on EST penetration through the human nipple (Figs. 3B and 4B). Keratin plug reduced EST flux by 5-fold in human nipple. After removing keratin plug, the flux and cumulative amount of EST was higher compared to human breast skin (Table 2). The amount of EST in the nipple was 3-fold high compared to the breast skin. Similar to 5FU, after removing the keratin plug, the permeation of EST through the porcine nipple was closer to the permeation of EST through human nipple. In time dependent penetration studies with porcine nipple in presence of keratin plug, very minimal drug was detected in the receptor medium after 6 hrs treatment. However with longer treatment, the drug penetrated across the nipple into the receptor medium (Figures S2 and S3 in S1 File). In presence of keratin plug, the drug retention in the nipple at the end of 6 hrs was similar for hydrophilic (5-FU) and lipophilic (EST) drugs. However, when the drug treatment was removed at 6 hrs and the study was continued for 48 hrs, relatively a higher amount of 5-FU diffused from the nipple into the receptor medium compared to EST (Figures S2 and S3 in S1 File). Taken together, the results from these studies indicate that the drug lipophilicity has a significant influence on permeation through the nipple.

### Characterization of transport pathways in nipple

**Figure 5:**
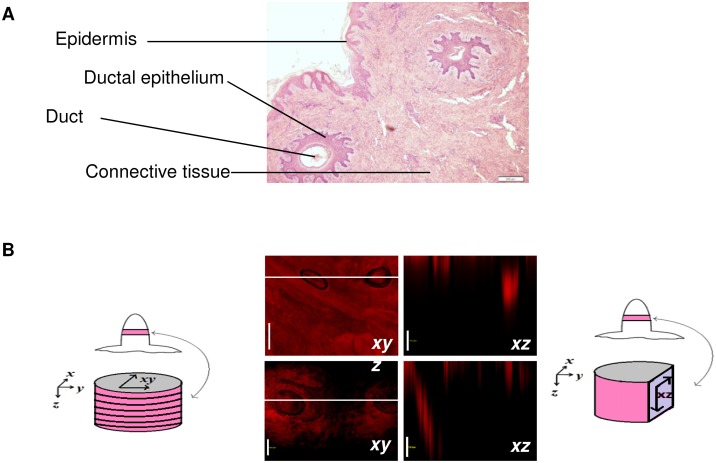
Confocal microscopic images of porcine nipple. (A) Cross section of porcine nipple after staining with hematoxylin-eosin. (Bar = 200 µm), (B) Confocal laser scanning microscopic images of porcine nipple, after treatment with hydrophilic dye sulforhodamine (SRB; upper panel) and hydrophobic dye Nile red (NR; lower panel). Image on the left panel is a cumulative xyz image of optical sections from 0 to 500 µm in the tissue. Images on the right panel are xz images from 0 to 500 µm (Bar = 100 µm).

**Figure 6:**
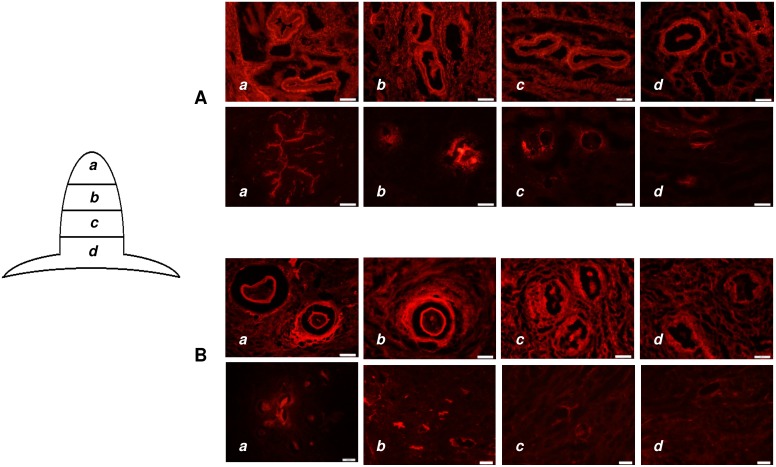
Fluorescence microscopic images of nipple. Fluorescence microscopic images of 7–8 µm thick cryosections of porcine (A) and human (B) nipple after treatment with the fluorescent dyes, SRB (upper panel in A and B) and NR (lower panel in A and B). Sections were taken from the entire length of the nipple starting from the tip to the base of the nipple. (Bar = 100 µm); SRB- sulforhodamine; NR- Nile red.

Fig. 5A shows the ducts in the nipple and the diameter of the ducts was around 100–150 µm. Confocal microscopy was used to visualize the transport pathway in the nipple (Fig. 5B). The hydrophilic dye, sulforhodamine was uniformly distributed in the ducts and the surrounding connective tissue. In contrast, the lipophilic dye, nile red was mainly localized to the ducts. To determine the dye distribution at different depths in the nipple, the tissue sections from the tip to the base of the nipple were visualized under a fluorescence microscope. As can be seen from Fig. 6A and 6B, the dye distribution pattern was consistent with the optical sections from confocal microscopy studies. The dye penetration was similar for both porcine and human nipple, which further suggests that porcine nipple could be a potential model for human nipple. Overall, the microscopy studies demonstrate that the duct is a major drug transport pathway to the underlying breast tissue, while the extent of distribution is influenced by the lipophilicity of the molecule.

### *In-vivo* biodistribution of 5-FU

**Figure 7:**
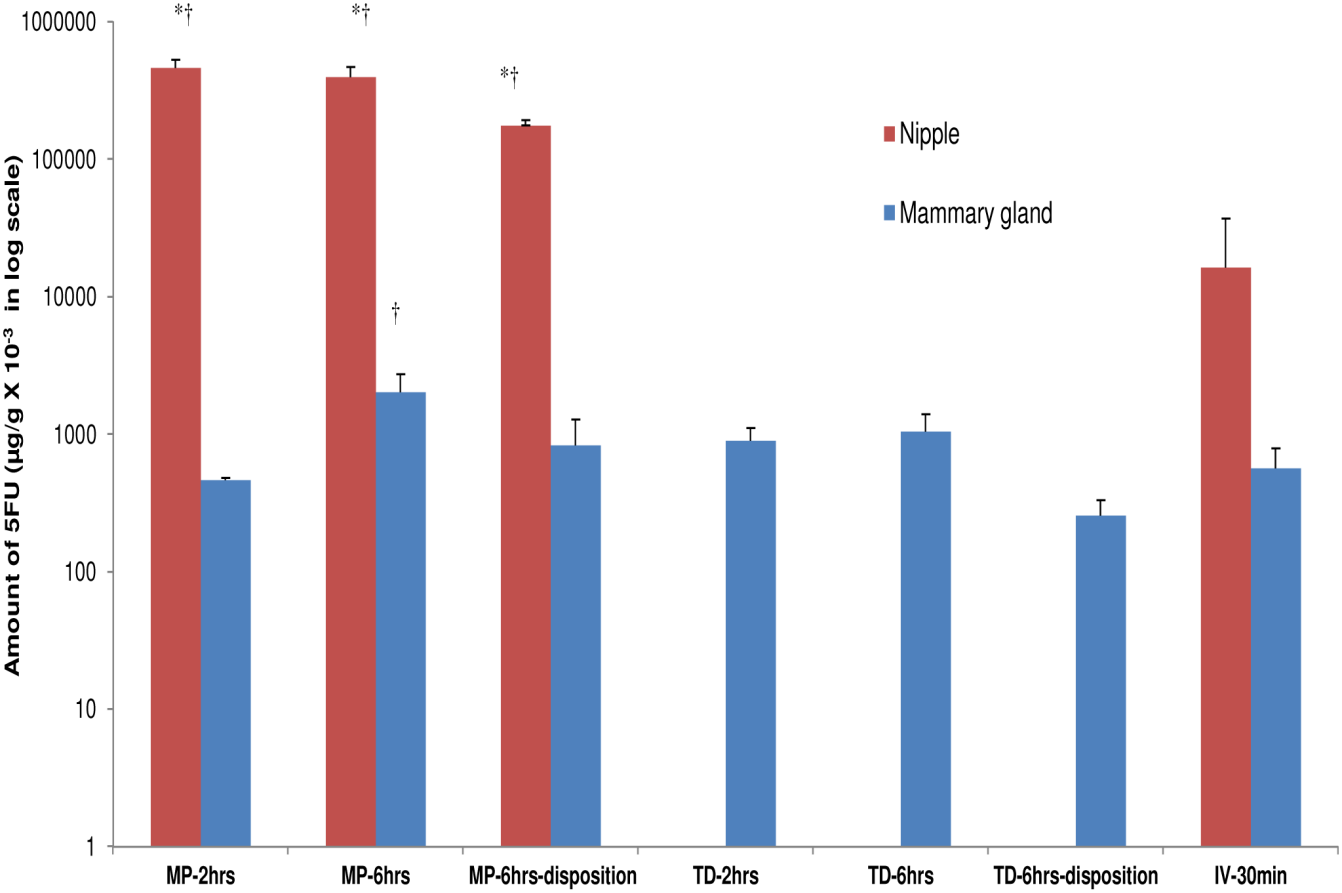
Biodistribution of 5-FU in rat breast tissue. Amount of 5-FU in rat mammary tissues after topical (nipple), transdermal (breast skin) and intravenous administration. Each value is represented as mean ± SD (n = 3); * is significant in comparison to transdermal treatment group; † is significant in comparison to intravenous injection group. The values are significant at p<0.05, by one-way ANOVA. MP- topical application on the mammary papilla (nipple); TD- transdermal delivery; IV-intravenous injection. The drug was applied on the mammary papilla or on the breast skin for 2 hrs or 6 hrs. In case of disposition studies, the drug was removed after 6 hrs treatment and the drug concentration was measured at 12 hrs.

**Figure 8:**
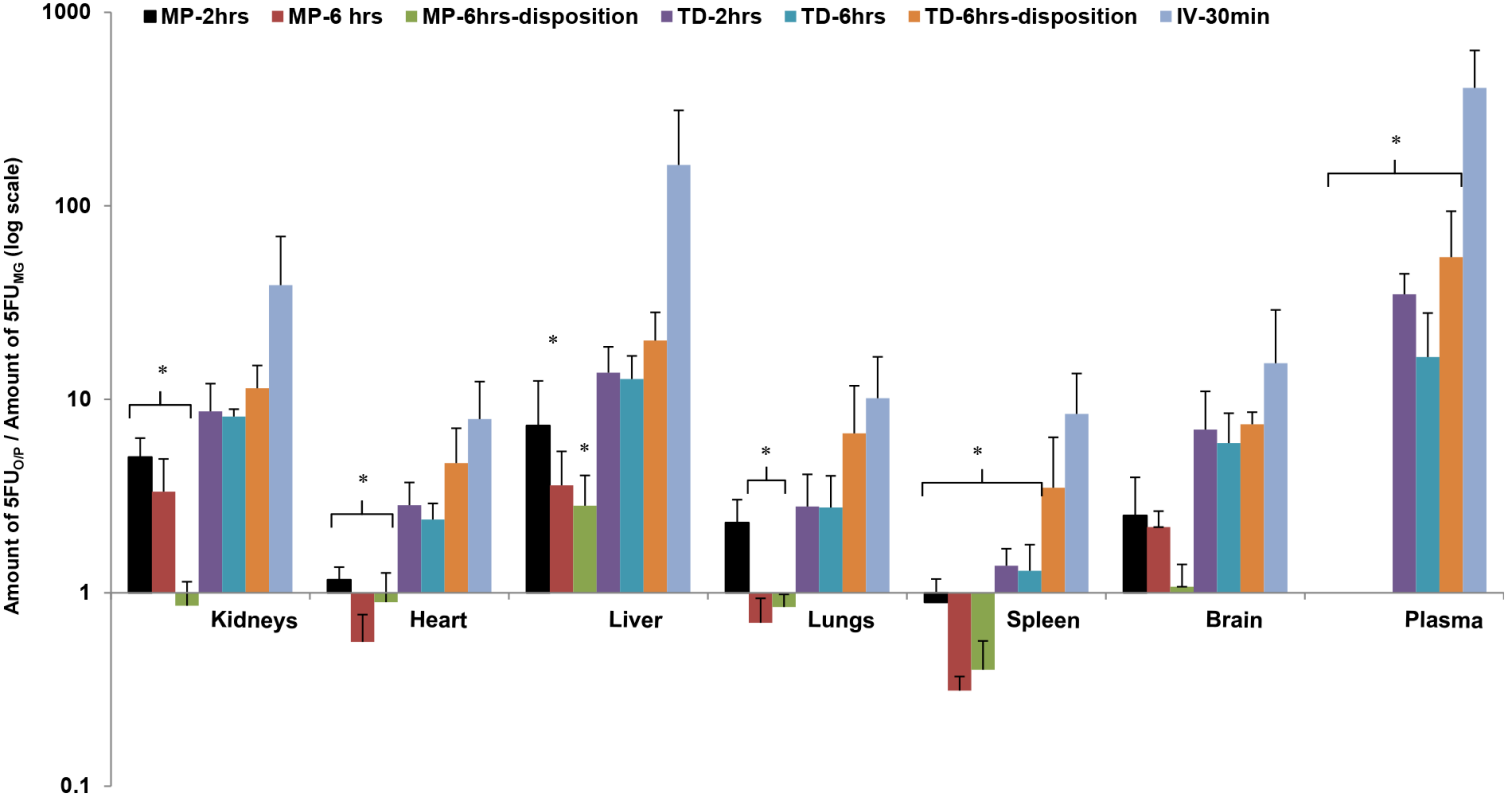
Biodistribution of 5-FU in plasma and organs in rat. Ratio of amount of 5-FU in rat organs or plasma (5-FU_O/P_) and amount of 5-FU in mammary gland (5-FU_MG_) after topical (nipple), transdermal (breast skin) and intravenous administration. Each value is represented as mean ± SD (n = 3); * is significant in comparison to intravenous injection group. The values are significant at p<0.05, by one-way ANOVA. MP- topical application on the mammary papilla (nipple); TD- transdermal delivery; IV-intravenous injection. The drug was applied on the mammary papilla or on the breast skin for 2 hrs or 6 hrs. In case of disposition studies, the drug was removed after 6 hrs treatment and the drug concentration in the plasma and organs were measured at 12 hrs.

Given that 5-FU is a drug used for breast cancer, it was chosen to demonstrate localized drug delivery in vivo in rats. In-vivo distribution of 5-FU was studied by topical application on the nipple or on the breast skin (transdermal), while intravenous 5-FU administration was used as a control. When applied on the nipple, the drug diffusion into the mammary gland increased with increase in treatment time (Fig. 7; Table S1 in S1 File). Most of the drug was retained in the nipple, which then slowly diffused into the mammary gland. This was evident from the disposition studies, where the drug retained in the nipple continued to diffuse into the mammary gland after the drug treatment was removed (Fig. 7). The treatment time did not significantly influence the drug penetration through the breast skin. As expected, there was no drug distribution into the nipple when the drug was applied on the breast skin (Fig. 7). Irrespecitve of the treatment time, the systemic drug distribution was lower after topical application compared to transdermal and intravenous treatment groups (Fig. 8). There was no detectable drug levels in the plasma after topical application (Fig. 8 and Table S1 in S1 File). On the other hand, transdermally delivered 5-FU was found to be distributed in plasma and other organs, but was lower compared to intravenous treatment group (Fig. 8). Overall, the results show that topical delivery through the nipple can achieve high drug concentration in the mammary gland with minimal systemic drug distribution.

## Discussion

Currently, there are no reliable methods to distinguish breast cancers that will develop into metastatic disease from those that will not. Therefore, all breast cancers are treated with aggressive therapies. Recently, it was reported that one to three deaths from overtreatment with aggressive therapies occur for every one breast cancer death avoided [21]. To this end, there is a strong need for developing safe and effective therapeutic approaches, especially for localized breast cancers. The transdermal delivery of anti-cancer agents through the breast skin is limited by the barrier properties of the stratum corneum [10], [11], [22], [23]. Pujol et al reported the transdermal delivery of 4-hydroxytamoxifen (4HT) in human subjects, but the drug levels in the breast tissue was 8–29 fold lower compared to oral drug administration [10]. Similarly, other studies have reported low drug concentrations in the breast after transdermal drug delivery through the breast skin [22], [23].

In contrast to skin, drug delivery through the nipple can overcome the skin barrier, resulting in direct drug delivery to the underlying breast tissue. The nipple has a thinner epidermis with multiple ducts and appendages, all of which results in higher drug penetration [14], [16], [17]. The time-dependent drug penetration through the nipple can be attributed to the formation of a drug depot in the nipple followed by slow diffusion of drug into the underlying breast tissue. Although the feasibility of topical delivery has been reported with porcine nipple [18], the results from our study for the first time demonstrate the feasibility of this delivery route in human nipple. Given the limited availability of human breast tissue and that only two nipple can be obtained from a single donor, it would be preferable to use a suitable animal model. To this end, the pig breast compares well with the human breast and the pig nipple has multiple duct openings similar to humans [24]. In non-lactating women, the ducts are blocked by a keratin plug [14], which can limit drug transport through the nipple. However, as demonstrated in this study, the keratin plug can be easily removed by wiping the nipple surface with 70% alcohol (Figure S1 in the S1 File). The human nipple has a larger keratin plug compared to the porcine nipple (Figure S1 in S1 File) which can be attributed to some of the differences on the effect of keratin plug between human and porcine nipple. However, after the keratin plug was removed, the drug penetration was comparable between porcine and human nipple (Figs. 3 and 4). Further, from the fluorescence microscopy studies, it is evident that the transport pathways are similar between porcine and human nipple (Fig. 6). In an earlier study by Lee et al [18], no detectable drug (hydrophobic anti-cancer molecules) was found in the receptor medium, possibly due to the presence of keratin plug. This is consistent with our results, where keratin plug limited drug transport through the nipple (Figs. 3 and 4; Figures S2 and S3 in S1 File). Taken together, the results suggest that drug delivery through the nipple is influenced by the drug’s lipophilicity. However, further studies are required to understand the influence of other physicochemical factors on drug delivery through the nipple.

The in-vivo results demonstrated the proof-of-principle for localized topical delivery through the nipple. Our results from i.v. injection are consistent with the short half-life reported for 5FU in rats [25]. The concentration of 5-FU in the breast after 6 hrs of topical application was 2–3 fold higher compared to transdermal and IV administration (Fig. 7 and Table S1 in S1 File). More importantly, the 5-FU level in the plasma after topical application was significantly lower compared to transdermal and i.v. administration (Fig. 8, Table S1 in S1 File). Unlike transdermal drug delivery through the skin, drug retained in the nipple can directly reach the underlying mammary gland. Komuro et al reported 3-fold higher drug concentration in the breast by iontophoretic delivery through the nipple compared to oral drug administration in dogs [26]. Further, they also showed that there was no detectable drug concentration in the plasma after topical delivery.

The 5-FU concentration (2 ng/mg) achieved in the mammary gland after 6 hrs of topical application is higher than the IC_50_ value reported for 5-FU in breast cancer cells (0.09–1.3 ng) [27], [28]. Recently, Stearns et al have shown that intraductal injection of 5-FU effectively reduces the tumor formation in rats compared to i.v. administration [8]. However, in contrast to topical delivery of 5-FU, the drug was rapidly cleared from the breast after intraductal injection, leading to systemic side effects. Taken together, the results from our proof-of-concept study suggest the possibility of achieving clinically relevant 5FU concentrations in the breast by topical delivery through the nipple. The topical delivery would be an attractive strategy for chemoprevention and treatment of pre-cancerous lesions in the breast. To this end, natural chemopreventive agents and low-dose chemotherapy (metronomic chemotherapy) are suitable for transpapillary delivery to the breast [29], [30]. Given the small area of drug application in the nipple, this route may be limited to the delivery of potent drug molecules. Since the drug is directly delivered to the breast, a lower dose may be required for topical delivery compared to systemic delivery. In addition, the drug penetration can be enhanced by altering drug concentration, vehicle composition and using penetration enhancers. Apart from simple formulations (lotions or gel), specialized delivery systems such as nipple pads or patches may be required for sustained drug delivery to the breast. However, it is important to ensure that the formulation is non-irritating to the skin. In summary, the design of appropriate formulations and delivery systems can lead to a safe and effective localized therapy for breast cancer.

## Conclusion

The findings from the study demonstrate that nipple is a potential route for direct drug delivery to the breast. The penetration through the nipple is influenced by the drug’s lipophilicity. The results demonstrate that porcine nipple can be used as an in-vitro model for human nipple, especially after removing the keratin plug. The results from the in-vivo animal studies showed that the topical delivery through nipple can achieve high drug concentration in the breast with minimal drug levels in the blood. Overall, the findings from the study can be used to develop localized therapeutic strategies for breast cancer and other breast diseases.

## Supporting Information

S1 File **Figures S1–S3 and Table S1.** Figure S1. Stereomicroscopic images of mammary papilla with and without keratin plug. Figure S2. Effect of treatment time on 5FU penetration through the porcine mammary papilla in presence of keratin plug. Figure S3. Effect of treatment time on EST penetration through the porcine mammary papilla in presence of keratin plug. Table S1. Concentration of 5-FU in plasma and various organs after different treatments.
(DOCX)

## References

[1] Jemal A, Bray F, Center MM, Ferlay J, Ward E, et al (2011) Global cancer statistics. CA: Cancer J Clin 61:69–90. 21296855 10.3322/caac.20107

[2] Virnig BA, Tuttle TM, Shamliyan T, Kane RL (2010) Ductal carcinoma in situ of the breast: a systematic review of incidence, treatment, and outcomes. J Natl Cancer Inst 102:170–178. 20071685 10.1093/jnci/djp482

[3] Thomsen A, Kolesar JM (2008) Chemoprevention of breast cancer. Am J Health-Syst Pharm 65:2221–2228. 19020189 10.2146/ajhp070663

[4] Avis NE, Crawford S, Manuel J (2005) Quality of life among younger women with breast cancer. J Clin Oncol 23:3322–3330. 15908646 10.1200/JCO.2005.05.130

[5] Wood AJ, Shapiro CL, Recht A (2001) Side effects of adjuvant treatment of breast cancer. N Eng J Med 344:1997–2008. 10.1056/NEJM200106283442607 11430330

[6] Darby SC, McGale P, Taylor CW, Peto R (2005) Long-term mortality from heart disease and lung cancer after radiotherapy for early breast cancer: prospective cohort study of about 300 000 women in US SEER cancer registries. Lancet Oncol 6:557–565. 16054566 10.1016/S1470-2045(05)70251-5

[7] Flanagan M, Love S, Hwang ES (2010) Status of intraductal therapy for ductal carcinoma in situ. Curr Breast Cancer Rep 2:75–82. 21124756 10.1007/s12609-010-0015-3 PMC2987566

[8] Stearns V, Mori T, Jacobs LK, Khouri NF, Gabrielson E, et al (2011) Preclinical and clinical evaluation of intraductally administered agents in early breast cancer. Sci Transl Med 3:106ra108. 10.1126/scitranslmed.3002368 PMC3616888 22030751

[9] Murata S, Kominsky SL, Vali M, Zhang Z, Garrett-Mayer E, et al (2006) Ductal access for prevention and therapy of mammary tumors. Cancer Res 66:638–645. 16423990 10.1158/0008-5472.CAN-05-4329

[10] Pujol H, Girault J, Rouanet P, Fournier S, Grenier J, et al (1995) Phase I study of percutaneous 4-hydroxy-tamoxifen with analyses of 4-hydroxy-tamoxifen concentrations in breast cancer and normal breast tissue. Cancer Chemotherap Pharmcol 36:493–498. 10.1007/BF00685799 7554041

[11] Mauvais-Jarvis P, Baudot N, Castaigne D, Banzet P, Kuttenn F (1986) Trans-4-hydroxytamoxifen concentration and metabolism after local percutaneous administration to human breast. Cancer Res 46:1521–1525. 3943109

[12] Lee O, Ivancic D, Chatterton RT Jr, Rademaker AW, Khan SA (2011) In vitro human skin permeation of endoxifen: potential for local transdermal therapy for primary prevention and carcinoma in situ of the breast. Breast Cancer: Targets and Therapy 3:61–70. 10.2147/BCTT.S20821 PMC3846656 24367176

[13] Sauvez F, Drouin DS, Attia M, Bertheux H, Forster R (1999) Cutaneously applied 4-hydroxytamoxifen is not carcinogenic in female rats. Carcinogenesis 20:843–850. 10334202 10.1093/carcin/20.5.843

[14] Rusby JE, Brachtel EF, Michaelson JS, Koerner FC, Smith BL (2007) Breast duct anatomy in the human nipple: three-dimensional patterns and clinical implications. Breast Cancer Res Treat 106:171–179. 17221150 10.1007/s10549-006-9487-2

[15] Kikuchi K, Tagami H, Akaraphanth R, Aiba S (2011) Functional analyses of the skin surface of the areola mammae: comparison between healthy adult male and female subjects and between healthy individuals and patients with atopic dermatitis. Br J Dermatol 164:97–102. 20874855 10.1111/j.1365-2133.2010.10076.x

[16] Shao C, Li A, Zhang J, Xue D, Zhang W (2012) Neglected aspect of the strategy for human breast diseases: Trans-areolar drug delivery. Med Hypoth 78:4–6. 10.1016/j.mehy.2011.09.027 21978968

[17] Love SM, Barsky SH (2004) Anatomy of the nipple and breast ducts revisited. Cancer 101:1947–1957. 15382093 10.1002/cncr.20559

[18] Lee LM, Davison Z, Heard CM (2010) In vitro delivery of anti-breast cancer agents directly via the mammary papilla (nipple). Int J Pharm 387:161–166. 20025946 10.1016/j.ijpharm.2009.12.021

[19] Rivera E, Chang JC, Semiglazov V, Burdaeva O, Kirby MG, et al (2014) Eniluracil plus 5-fluorouracil and leucovorin: treatment for metastatic breast cancer patients in whom capecitabine treatment rapidly failed. Clin Breast Cancer 14:26–30. 24183612 10.1016/j.clbc.2013.08.018

[20] Goodsell DS (2002) The molecular perspective: tamoxifen and the estrogen receptor. Stem Cells 20:267–268. 12004085 10.1634/stemcells.20-3-267

[21] Baum M (2013) Harms from breast cancer screening outweigh benefits if death caused by treatment is included. BMJ 346:f385. 23344314 10.1136/bmj.f385

[22] Miller JA, Thompson PA, Hakim IA, Lopez AM, Thomson CA, et al (2012) Safety and feasibility of topical application of limonene as a massage oil to the breast. J Cancer Therap 3:749–754. 10.4236/jct.2012.325094 PMC3824622 24236248

[23] Güngör S, Delgado-Charro MB, Masini-Etévé V, Potts RO, Guy RH (2013) Transdermal flux predictions for selected selective oestrogen receptor modulators (SERMs): Comparison with experimental results. J Control Release 172:601–606. 24076520 10.1016/j.jconrel.2013.09.017

[24] Deakin A (1936) Induction of Mammary Ducts. Nature 137:619–620.

[25] Celio LA, DiGregorio GJ, Ruch E, Pace JN, Piraino AJ (1983) 5-Fluorouracil concentrations in rat plasma, parotid saliva, and bile and protein binding in rat plasma. J Pharm Sci 72:597–599. 6875817 10.1002/jps.2600720605

[26] Komuro M, Suzuki K, Kanebako M, Kawahara T, Otoi T, et al (2013) Novel iontophoretic administration method for local therapy of breast cancer. J Control Release 168:298–306. 23562634 10.1016/j.jconrel.2013.03.021

[27] Hernandez-Vargas H, Ballestar E, Carmona-Saez P, von Kobbe C, Banon-Rodriguez I, et al (2006) Transcriptional profiling of MCF7 breast cancer cells in response to 5-Fluorouracil: relationship with cell cycle changes and apoptosis, and identification of novel targets of p53. Int J Cancer 119:1164–1175. 16557594 10.1002/ijc.21938

[28] Li X, Kong X, Kong X, Wang Y, Yan S, et al (2013) 53BP1 sensitizes breast cancer cells to 5-fluorouracil. PLoS One 8:e74928. 24040364 10.1371/journal.pone.0074928 PMC3765449

[29] Chun YS, Bisht S, Chenna V, Pramanik D, Yoshida T, et al (2012) Intraductal administration of a polymeric nanoparticle formulation of curcumin. Carcinogenesis 33:2242–2249. 22831956 10.1093/carcin/bgs248 PMC3584967

[30] Kaur H, Budd GT (2004) Metronomic therapy for breast cancer. Curr Oncol Rep 6:49–52. 14664761 10.1007/s11912-996-0009-5

