# Supplementary material for: Transpapillary Drug Delivery to the Breast
Source: PLoS One. 2014 Dec 29;9(12):e115712. doi: 10.1371/journal.pone.0115712 (PMC4278765; doi:10.1371/journal.pone.0115712)
Supplement: S1 File — Figures S1–S3 and Table S1. Figure S1. Stereomicroscopic images of mammary papilla with and without keratin plug. Figure S2. Effect of treatment time on 5FU penetration through the porcine mammary papilla in presence of keratin plug. Figure S3. Effect of treatment time on EST penetration through the porcine mammary papilla in presence of keratin plug. Table S1. Concentration of 5-FU in plasma and various organs after different treatments. (DOCX) [file pone.0115712.s001.docx]

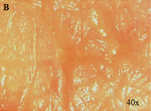


**40x**

**A**


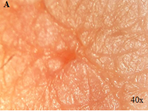


**B**

**40x**


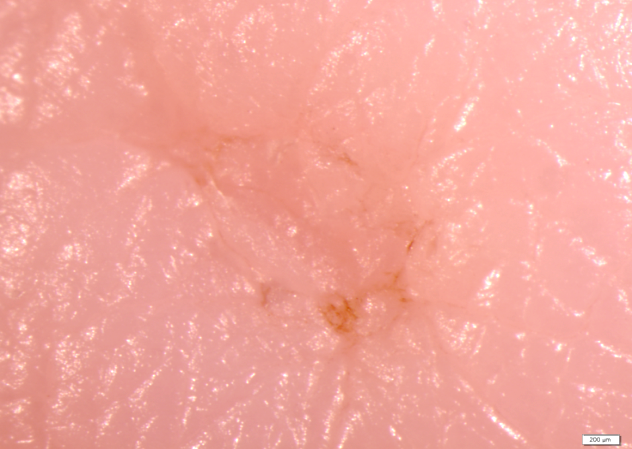

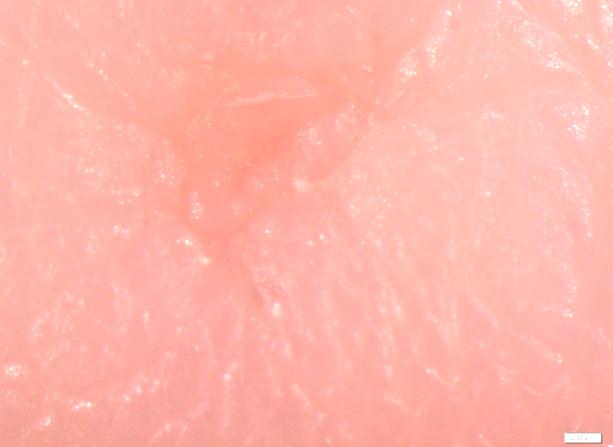


**A**

**B**

**Figure S1.** **Keratin plug removal from excised mammary papilla**. Stereomicroscopic images of porcine (upper panel) and human (lower panel) mammary papilla before (A) and after (B) keratin plug removal with 70% alcohol swipe. (Bar= 200 μm)


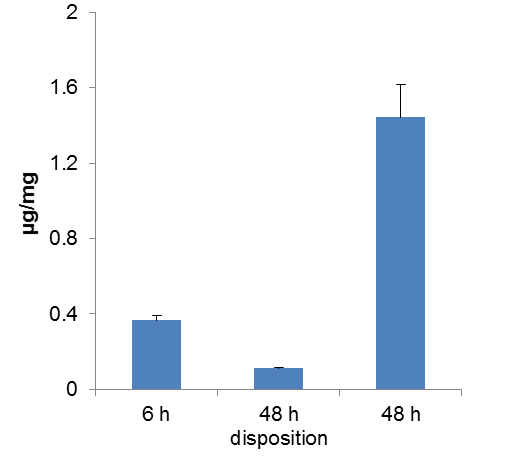

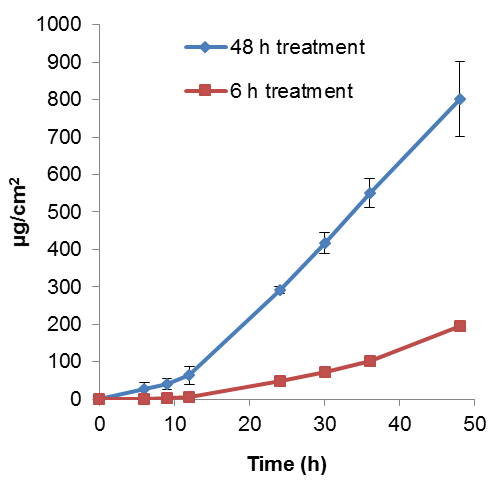


*

*

*

**A**

**B**

**Figure S2.** **Effect of treatment time on 5FU penetration through the porcine mammary papilla in presence of keratin plug**. (A) *In vitro* permeation of 5FU through porcine mammary papilla after treatment for 6 hrs and 48 hrs. In case of 6 hrs study, the treatment was removed after 6 hrs and the drug disposition was measured till 48 hrs. No drug was detected in the receptor medium at the end of 6hrs. (B) retention of 5FU in porcine mammary papilla after 6 hrs treatment, 6 hrs treatment followed by 48 hrs disposition study and 48 hrs drug treatment. Each data point is represented as mean ± SD (*n*= 3). Data is significant at *p*<0.05.


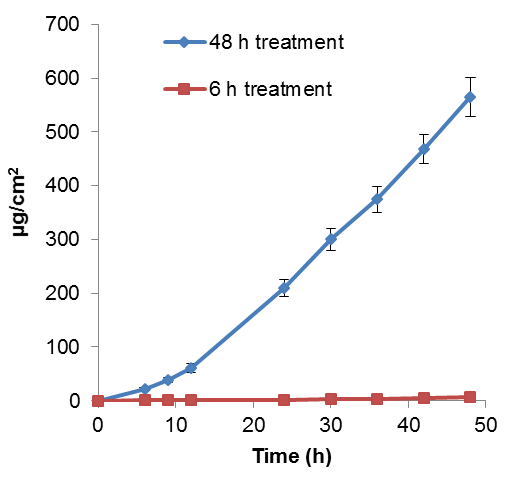

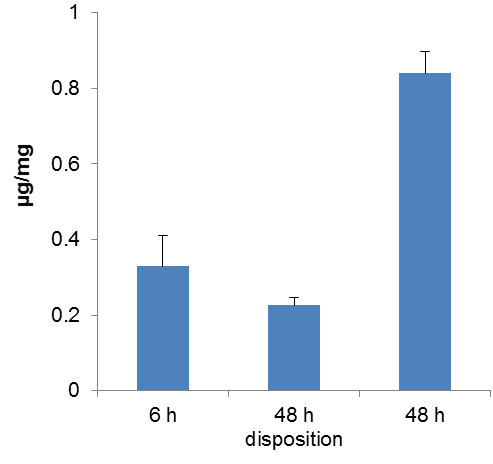


*

**A**

**B**

**Figure S3.** **Effect of treatment duration on EST penetration via porcine mammary papilla**. (A) *In vitro* permeation of EST through porcine mammary papilla after treatment for 6 hrs and 48 hrs. In case of 6 hrs study, the treatment was removed after 6 hrs and the drug disposition was measured till 48 hrs. No drug was detected in the receptor medium at the end of 6hrs. (B) retention of EST in porcine mammary papilla after 6 hrs treatment, 6 hrs treatment followed by 48 hrs disposition study and 48 hrs drug treatment. Each data point is represented as mean ± SD (*n*= 3). Data is significant at *p*<0.05.

**Table S1.** Concentration of 5-FU in plasma and various organs in rat after different treatments.

|  | **Mammary gland** | **Nipple** | **Kidneys** | **Heart** | **Liver** | **Lungs** | **Spleen** | **Brain** | **Plasma** |
| --- | --- | --- | --- | --- | --- | --- | --- | --- | --- |
| **MP-2 hrs** | 0.463 ± 0.015^b^ | 460.784 ± 69.054^cdefg^ | 0.340 ± 0.066^bdeg^ | 0.208 ± 0.046^deg^ | 0.134 ± 0.071^g^ | 0.323 ± 0.197 | 0.295 ± 0.073^deg^ | 0.241 ± 0.093 | ND^deg^ |
| **MP-6 hrs** | 2.036 ± 0.698^cdfg^ | 395.362 ± 70.469^cdefg^ | 0.893 ± 0.203^c^ | 0.397 ± 0.122^de^ | 0.284 ± 0.088^g^ | 0.380 ± 0.141 | 0.418 ± 0.066^g^ | 0.936 ± 0.252 | ND^deg^ |
| **MP-6 hrs-disposition** | 0.836 ± 0.438 | 175.991 ± 16.380^defg^ | 0.096 ± 0.018^deg^ | 0.275 ± 0.105^de^ | 0.087 ± 0.023^g^ | 0.190 ± 0.027^de^ | 0.240 ± 0.053^deg^ | 0.182 ± 0.010 | ND^deg^ |
| **TD-2 hrs** | 0.895 ± 0.214 | ND | 1.069 ± 0.249^f^ | 0.792 ± 0.022^fg^ | 0.416 ± 0.032^g^ | 0.540 ± 0.114 | 0.706 ± 0.224 | 1.034 ± 0.662 | 0.454 ± 0.135^efg^ |
| **TD-6 hrs** | 1.050 ± 0.351 | ND | 1.230 ± 0.180^f^ | 0.797 ± 0.085^fg^ | 0.454 ± 0.046^g^ | 0.604 ± 0.080 | 0.722 ± 0.074^f^ | 1.149 ± 0.719 | 0.229 ± 0.131^g^ |
| **TD-6 hrs-disposition** | 0.256 ± 0.074 | ND | 0.375 ± 0.066^g^ | 0.314 ± 0.111 | 0.164 ± 0.021^g^ | 0.310 ± 0.132 | 0.350 ± 0.163^g^ | 0.311 ± 0.138 | 0.169 ± 0.056^g^ |
| **IV-30 min** | 0.563 ± 0.226 | 16.346 ± 20.714 | 1.093 ± 0.348 | 0.466 ± 0.170 | 1.083 ± 0.466 | 0.318 ± 0.101 | 0.947 ± 0.146 | 0.529 ± 0.156 | 1.241 ± 0.072 |

Each value is represented as mean ± SD (n= 3); ‘b’ is significant in comparison to topical treatment via nipple for 6 hrs; ‘c’ is significant in comparison to 6 hrs after topical treatment via nipple for 6 hrs; ‘d’ is significant in comparison to transdermal treatment for 2 hrs; ‘e’ is significant in comparison to transdermal treatment for 6 hrs; ‘f’ is significant in comparison to 6 hrs after transdermal treatment for 6 hrs; ‘g’ is significant in comparison to 30 minutes after intravenous injection. The values are significant at p<0.05. MP-topical application on the mammary papilla (nipple); TD-transdermal application on the breast skin; IV-intravenous injection, ND-not detected. The drug was applied for 2 to 6 hrs and for the disposition studies, the drug application was removed after 6 hrs and the study was continued for further 6 hrs.
